# Non-coordinating charge transfer enables ultrafast desolvation of hydrated zinc ions in the outer Helmholtz layer for stable aqueous Zn metal batteries

**DOI:** 10.1093/nsr/nwaf070

**Published:** 2025-02-22

**Authors:** Xiuli Guo, Qiaoling Peng, Rui Yang, Gengyou Cao, Jianfeng Wen, Kyungsoo Shin, Ye Zheng, Sarayut Tunmee, Caineng Zou, Yongping Zheng, Xiaolong Zhou, Yongbing Tang

**Affiliations:** Advanced Energy Storage Technology Research Center, Shenzhen Institutes of Advanced Technology, Chinese Academy of Sciences, Shenzhen 518055, China; Advanced Energy Storage Technology Research Center, Shenzhen Institutes of Advanced Technology, Chinese Academy of Sciences, Shenzhen 518055, China; Nano Science and Technology Institute, University of Science and Technology of China, Suzhou 215123, China; Advanced Energy Storage Technology Research Center, Shenzhen Institutes of Advanced Technology, Chinese Academy of Sciences, Shenzhen 518055, China; Advanced Energy Storage Technology Research Center, Shenzhen Institutes of Advanced Technology, Chinese Academy of Sciences, Shenzhen 518055, China; Nano Science and Technology Institute, University of Science and Technology of China, Suzhou 215123, China; Advanced Energy Storage Technology Research Center, Shenzhen Institutes of Advanced Technology, Chinese Academy of Sciences, Shenzhen 518055, China; Shenzhen College of Advanced Technology, University of Chinese Academy of Sciences, Shenzhen 518055, China; Advanced Energy Storage Technology Research Center, Shenzhen Institutes of Advanced Technology, Chinese Academy of Sciences, Shenzhen 518055, China; Shenzhen College of Advanced Technology, University of Chinese Academy of Sciences, Shenzhen 518055, China; Advanced Energy Storage Technology Research Center, Shenzhen Institutes of Advanced Technology, Chinese Academy of Sciences, Shenzhen 518055, China; Nano Science and Technology Institute, University of Science and Technology of China, Suzhou 215123, China; Synchrotron Light Research Institute (Public Organization), Nakhon Ratchasima 30000, Thailand; PetroChina Shenzhen New Eergy Research Institute Co., LTD., Shenzhen 518054, China; Advanced Energy Storage Technology Research Center, Shenzhen Institutes of Advanced Technology, Chinese Academy of Sciences, Shenzhen 518055, China; Shenzhen College of Advanced Technology, University of Chinese Academy of Sciences, Shenzhen 518055, China; Advanced Energy Storage Technology Research Center, Shenzhen Institutes of Advanced Technology, Chinese Academy of Sciences, Shenzhen 518055, China; Shenzhen College of Advanced Technology, University of Chinese Academy of Sciences, Shenzhen 518055, China; Advanced Energy Storage Technology Research Center, Shenzhen Institutes of Advanced Technology, Chinese Academy of Sciences, Shenzhen 518055, China; Shenzhen College of Advanced Technology, University of Chinese Academy of Sciences, Shenzhen 518055, China

**Keywords:** Fermi-level engineering, non-coordinating charge transfer, fast desolvation, nitrogen-doped amorphous carbon, aqueous Zn metal batteries

## Abstract

The formation of a strong coordination structure, [Zn(H_2_O)_6_]^2+^ often increases direct contact between the solvated H_2_O and Zn anodes in the inner Helmholtz layer, which exacerbates undesirable side reactions and dendrite growth, hindering the practical application of aqueous Zn metal batteries. Here, we show that the solvated H_2_O can be effectively minimized by an artificial solid electrolyte interphase (SEI) consisting of highly nitrogen-doped amorphous carbon (NC) and perfluorosulfonic acid polymer (Nafion). Theoretical and experimental analyses reveal that NC raises the Fermi level of the composite SEI and activates the non-coordinating charge transfer from the SEI to [Zn(H_2_O)_6_]^2+^, which leads to ultrafast desolvation of hydrated Zn-ions in the outer Helmholtz layer; while the Nafion framework ensures selective transport channels for Zn ions. Remarkably, the derived NC-Nafion@Zn symmetric cell exhibits a long lifespan (3400 h, 1 mA cm^–2^; 2000 h, 5 mA cm^–2^); moreover, the NC-Nafion@Zn//Mn_4_O_3_-carbon nanotubes full battery delivers ultralong cycling stability of 9300 cycles at 2 A g^–1^ with a high retention of 91.3%.

## Teaser

An artificial solid electrolyte interphase (SEI) with upshifting Fermi level and cation screening channel enables ultrafast desolvation of [Zn(H_2_O)_6_]^2+^ in the outer Helmholtz layer through non-coordinating charge transfer process, realizing a robust Zn anode.

## INTRODUCTION

Rechargeable aqueous Zn metal batteries (AZMBs) are considered one of the promising candidates for grid-scale energy storage due to their inherent safety, satisfying energy and power density, abundant resources, and environmental friendliness [1–4]. Unfortunately, the Zn anode has a high thermodynamic instability in aqueous solutions due to the low redox potential (−0.76 V vs. standard hydrogen electrode), and worse, the divalent Zn^2+^ cation is strongly solvated by nucleophilic H_2_O as [Zn(H_2_O)_6_]^2+^, which carries the solvated H_2_O into the inner Helmholtz layer (IHL) where it comes into direct contact with the Zn metal, thus exacerbating undesirable side reactions associated with the solvated H_2_O (eg., hydrogen evolution reaction (HER), corrosion, and passivation). The sluggish desolvation kinetics of [Zn(H_2_O)_6_]^2+^ also inhibit the smooth Zn deposition, resulting in uncontrolled growth of Zn dendrites [5–8]. Analogous to the limitations caused by lithium dendrites [9–11], the fatal formation of zinc dendrites seriously reduce the Coulombic efficiency (CE) and shorten the cycle life of AZMBs, hindering their practical development [12–15]. To address these, it is imperative to develop a strategy that can accelerate the desolvation process of [Zn(H_2_O)_6_]^2+^ outside the IHL, thus reducing the direct contact between solvated H_2_O and the Zn anode, as well as providing a uniform flux of Zn^2+^ for homogeneous Zn deposition.

Hitherto, the widely used strategies to minimize the solvated H_2_O can be categorized into electrolyte engineering [16–19], and Zn anode interfacial modification [10,20–22]. In the former strategy, a high-concentration electrolyte (HCE) [23–25] and strongly coordinating additives [26–29] are typically used to reduce the coordinating number of H_2_O in the solvation sheath, however, either the increased viscosity of HCE or the strong interaction between additives and Zn^2+^ will again bring about the issue of sluggish kinetics. Alternatively, the construction of an artificial solid electrolyte interphase (SEI) can not only isolate solvent water but also disrupt the interaction between H_2_O and Zn^2+^ by size-limited ion channels or strong polar functional groups [30–34]. For example, Lan *et al*. [31], designed a series of covalent organic frameworks (COFs) as Zn anode protective layer. The zinc affinity (zincophilic) polar functional groups (C=N, –C=O) in COFs can coordinate with Zn^2+^ ions and promote the removal of hydrated molecules of [Zn(H_2_O)_6_]^2+^, achieving the reduction of HER and uniform Zn^2+^ flux. Despite the inspiring achievements, size-limited ion channels or the strongly coordinating ability of the current SEI will slow down the diffusion kinetics of Zn^2+^ ions, which is not conducive to the rate performance of AZMBs [35–37].

Given the above considerations, the design of an artificial SEI layer with broad ion channels and non-coordinating charge transfer capability is essential to facilitate the rapid desolvation of [Zn(H_2_O)_6_]^2+^ and uniform Zn deposition without hampering ion transport. As a proof of concept, the produced artificial SEI layer consisting of highly nitrogen-doped amorphous carbon (NC) and perfluorosulfonic acid polymer (Nafion), in which Nafion acts as a cation-selective membrane that can selectively shield anions and provide sufficient space for the transport of Zn ions; while the high nitrogen doping concentration in NC can upshift the Fermi-level and activate the dehydration of [Zn(H_2_O)_6_]^2+^ in the outer Helmholtz layer (OHL) through non-coordinating charge transfer, which minimizes the solvated H_2_O and promotes uniform Zn deposition. Eventually, benefiting from the synergistic effect of high-Fermi-level NC and cation selective Nafion composite artificial SEI layer, the NC-Nafion@Zn symmetric battery delivers a long lifespan (3400 h, 1 mA cm^–2^; 2000 h, 5 mA cm^–2^) and a high CE of plating/stripping (99.1%). Furthermore, the NC-Nafion@Zn//Mn_4_O_3_-carbon-nanotubles (CNTs) full battery shows a high capacity of 116.7 mAh g^–1^ after 9300 cycles at 2 A g^–1^ with a retention of 91.3%. This work provides a new perspective on achieving stable Zn anodes and facilitates the practical development of AZMBs.

## RESULTS AND DISCUSSION

**Figure 1.**
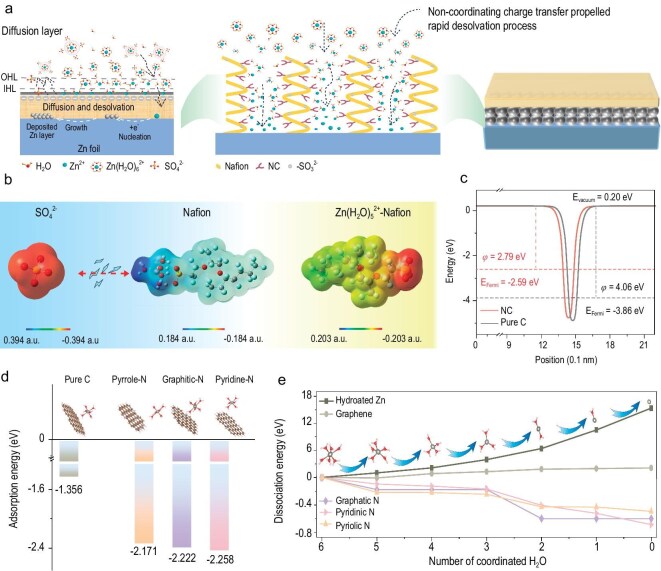
(a) Schematic diagram of the Zn deposition on NC-Nafion@Zn anode. (b) MEP distribution of SO_4_^2−^, Nafion and Zn(H_2_O)_5_^2+^-Nafion. (c) The Fermi level and work function of NC and pure C. (d) The adsorption energy of Zn(H_2_O)_6_^2+^ cluster on pure C and NC materials. (e) The dissociation energy of Zn(H_2_O)_6_^2+^ cluster in various chemical environments.

The strategy diagram of NC-Nafion artificial SEI structural design is shown in Fig. 1a. The chemically inert protective layer of Nafion ionomer can serve as a Zn^2+^ ion conductor, as well as isolate solvent water molecules and shield anions to eliminate side reactions. The evenly decorative high-Fermi-level NC in Nafion accelerates the decoupling process of [Zn(H_2_O)_6_]^2+^ outside the inner Helmholtz plane by reducing the charge density of Zn^2+^, achieving fast desolvation and charge transfer kinetics. To verify the working mechanism of NC-Nafion artificial SEI, theoretical analyses were first conducted based on density functional theory (DFT). As can be seen from the molecular electrostatic potential (MEP) distribution maps in Fig. 1b, the red area represents electron concentration and the blue area is the electron-poor area. It is easier to conclude that the sulfate ion in the electrolyte and the sulfonic acid group of the Nafion chain exhibit strong mutual exclusion due to their electron-rich structures. While for [Zn(H_2_O)_5_]^2+^, the sulfonic acid group can serve as a capture gripper for regulating their rapid directional transport. Therefore, the channel with identifiable ion sieving function of Nafion chains can selectively block the transport of sulfate ions and enhance the transport kinetics of hydrated Zn^2+^, which will reduce the generation of Zn_4_SO_4_(OH)_6_·H_2_O (ZSH) by-products and Zn dendrites.

However, a large number of coordinated water molecules would pass through the pure Nafion membrane due to its spacious channel size (∼4 nm) [38], which causes side reactions between the Zn substrate and active solvent water. Thereupon, the introduction of electron-rich high Femi-level NC in Nafion is necessary for our strategy. As shown in Fig. 1c, the NC and presence of the N element in NC raises the Fermi level as compared with the pure C materials (‒2.59 and ‒3.86 eV vs. vacuum). Their density-of-state calculation results are displayed in Fig. S1. Moreover, NC showcases a lower work function than that of pure C, demonstrating an enhanced electron-donating ability [39,40], which also reveals that the presence of abundant N-dope structures upshifts the Fermi level and will make the electron transport easier from NC to Zn^2+^.

Furthermore the calculations show that the NC with different N-doping types, such as graphitic-N, pyrrolic-N, and pyridinic-N, exhibit remarkable negative adsorption energy for Zn(H_2_O)_6_^2+^ (‒2.17∼ ‒2.26 eV, Fig. 1d), which is significantly stronger than the pure C (‒1.36 eV), indicating enhanced interaction between Zn(H_2_O)_6_^2+^ ions and NC. Then the dissociation energy is calculated to further quantify the easier dissociation process of [Zn(H_2_O)_6_]^2+^, as exhibited in Fig. 1e. The [Zn(H_2_O)_6_]^2+^ shows high dissociation energy on bare Zn (1.01–15.36 eV) and pure C (‒0.11–2.09 eV), indicating the difficult and sluggish desolvation processes. While on the NC, a spontaneous desolvation process can be observed due to the negative dissociation energy (‒0.09 to ‒0.69 eV). The specific dissociation energies on various substrates are shown in Table S1. The desolvation process of the adsorbed Zn(H_2_O)_6_^2+^ on pure C and different N-doped carbon sites are shown in Fig. S2 and Videos 1–3, respectively. Notably, there is no formation of any type of chemical bonds between the Zn^2+^ and NC, suggesting that the desolvation process of Zn(H_2_O)_6_^2+^occurs in the OHL and eliminates the water-reduced side reactions. Differing from the previous reports about the formation of new Zn^2+^ solvation structures through the coordination interaction with other species [31,32,41–46], our design provides a new approach to eliminate coordinated water without coordination bonds, which will conserve the re-dissociation step during ion transport, greatly accelerating the charge transfer kinetics.

Then, the barde charge evolution of hydrated Zn^2+^ during the diffusion process is shown in Fig. S3. The barde charge of Zn^2+^ in an individual [Zn(H_2_O)_6_]^2+^ cluster is 10.56 eV, as they pass through the NC-Nafion layer, the values increase sharply, which will lead to the decrease of charge density and a decline of control over the solvent shell molecules. That is to say, the hydrated Zn^2+^ ions are completely reduced and dehydrated as they reach the near anode interface, realizing fast charge transfer dynamics and elimination of water-related side reactions. As a consequence, the design of the NC-Nafion artificial SEI layer has great potential to integrate the multifunctional advantages of ion sieving, capture, desolvation, and uniform deposition for Zn metal anodes. During the transmission process of Zn(H_2_O)_6_^2+^ in NC-Nafion artificial SEI, it can gradually dehydrate/dissociate by obtaining electrons from NC, and finally achieving complete dehydration and undergoing a reduction reaction at the interface of the NC-Nafion and Zn substrate, resulting in elimination of active water induced side reactions and achieving homogeneous nucleation and deposition of Zn^2+^ ions.

**Figure 2.**
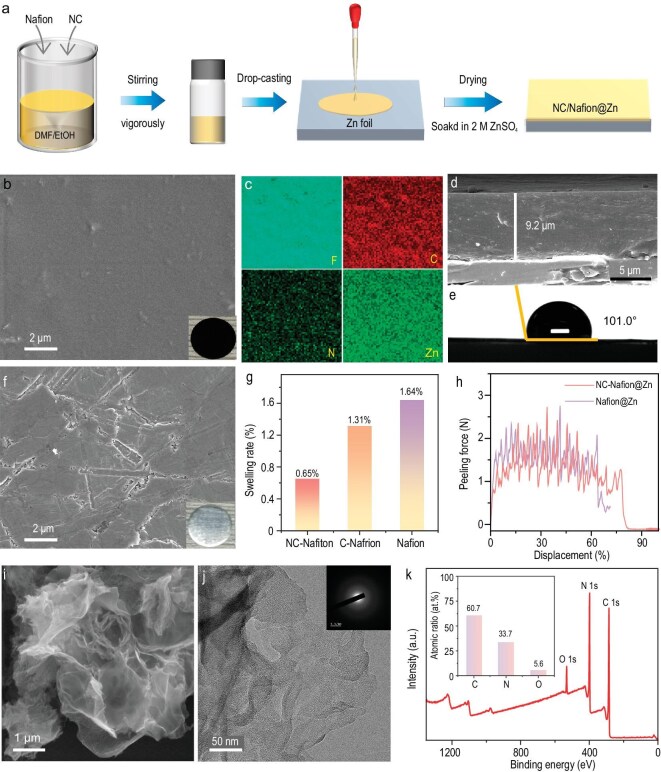
(a) Schematic diagram of the preparation of NC-Nafion@Zn electrode. (b) SEM and digital pictures (inset), (c) corresponding EDS mappings, (d) cross-sectional SEM image, and (e) contact angle of NC-Nafion@Zn. (f) SEM image of bare Zn. Inset shows the corresponding optical picture. (g) The swelling rates of NC-Nafion, C-Nafion, and Nafion membranes. (h) The peeling performance of NC-Nafion@Zn and Nafion@Zn electrodes. (i) SEM image, (j) TEM and SAED pattern of NC powders. (k) XPS survey spectra of NC. The inset is the specific value of C, N, and O atomic ratios.

To confirm the above calculation conclusion, the artificial SEI of NC-Nafion was fabricated by a drip coating procedure, as illustrated in Fig. 2a. The detailed preparation process is presented in the experimental section in the Supporting Information (SI). The surface morphology and composition of NC-Nafion interphase were characterized by scanning electron microscopy (SEM) and energy dispersive spectroscopy (EDS) mapping (Fig. 2b–d). It can be noticed that the smooth NC-Nafion layer, with a thickness of 9.2 μm, is tightly adhered to the surface of the Zn substrate, and the N, C, and F elements were evenly distributed through the entire interphase. To showcase the advantages of NC-Nafion@Zn in the desolvation of [Zn(H_2_O)_6_]^2+^ and charge transfer dynamics, pure carbon material modified Nafion and Nafion coated Zn anodes (named as C-Nafion@Zn and Nafion@Zn) were obtained through similar preparation processes. The C-Nafion and Nafion layers show uniform appearances with thicknesses of 9.6 and 9.3 μm (Fig. S4a, b and Fig. S5a, b). Bare Zn shows a tough surface texture (Fig. 2f). Moreover, the hydrophobicity between various electrodes and 2 M ZnSO_4_ of electrolyte represents a significant factor influencing the Zn dendrites and side reactions [47]. The obtained anodes display similar hydrophobicity according to their contact angles, that of NC-Nafion@Zn is 101° (Fig. 2e), and the C-Nafion@Zn, Nafion@Zn, and bare Zn anodes are 110, 100.7, and 101.8°, respectively (Figs S4c, S5c, and S6). However, when soaking NC-Nafion, C-Nafion, and Nafion layers in water for 48 h at room temperature, the NC-Nafion membrane shows the smallest water uptake ratio (0.65%, Fig. 2g), suggesting that the composite coatings possess lower swelling effects and improved water blocking ability, which may help to reduce side reactions and achieve long-term cycling stability. The X-ray diffraction (XRD) measurement results reveal that there is no additional diffraction peak after establishing the NC-Nafion, C-Nafion, and Nafion layers on the Zn foil (Fig. S7).

For the NC-Nafion layer, the decorated NC with a high Fermi-level is a crucial element for the decoupling of [Zn(H_2_O)_6_]^2+^ clusters. Therefore, it is necessary to acquire the morphology and structural information of NC. The SEM in Fig. 2i reveals that the NC powders exhibit a flowerlike architecture stacked by ultra-thin nano-sheets, which is beneficial for exposing more active sites to promote the desolvation process. The pure carbon material exhibits a layered-stacking appearance (Fig. S8). The transmission electron microscope (TEM) and the selected area electron diffraction (SAED) pattern in Fig. 2j show the thin sheet appearance and amorphous structure of NC, and the result can also be confirmed by its broad and weak XRD peak (Fig. S9). The high-angle angular dark field (HAADF) image and corresponding EDS mappings display the uniform distribution of doped N elements in NC (Fig. S10). The Raman spectrum of NC in Fig. S11 exhibits two typical bands of D peak (disorder or defect structures) and G peak (graphitic structures) at 1361 and 1587 cm^–1^. The related intensity ratio of *I_D_*/*I_G_* is ∼1.33, indicating abundant defects and low graphite proportion [48–51]. The XPS full spectrum in Fig. 2k reveals the presence of the C, N, and O elements, and the content of the N atom is as high as 33.7 at.%. Moreover, the CHN elemental analysis determines the similar content of N element (32.5 at.%). The high amount of doping N atoms in NC can act as electron donors to increase the density of free charge carriers, which helps to promote the solvation process of hydrated Zn^2+^ and reduce water-induced side reactions (discussed in later sections). The high-resolution N 1 *s* spectrum (Fig. S12) was deconvoluted into pyridinic-N (398.1 eV), pyrrolic-N (399.7 eV), and graphitic-N (400.6 eV) peaks, respectively [52]. In the C 1 *s* spectrum (Fig. S13), the characteristic peaks at 284.8, 285.8, 286.9, and 288.6 eV correspond to C–C, C–N, C–O, and C=O bonds [53,54].

**Figure 3.**
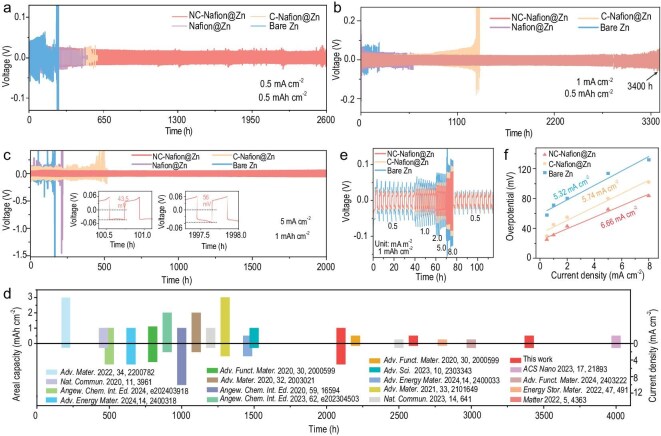
Battery performances of different symmetrical cells: cycling stability at (a) 0.5 mA cm^−2^ and 0.5 mAh cm^−2^, (b) 1 mA cm^−2^ and 0.5 mAh cm^−2^, (c) 5 mA cm^−2^ and 1 mAh cm^−2^, and corresponding voltage profiles at different cycles, respectively; (d) cyclability comparison with recent works; (e) rate performances; (f) the *i_0_* values of different electrodes.

To elucidate the superiority of the NC-Nafion layer on the cycling lifespan of Zn plating/stripping, the cyclability of symmetric Zn||Zn cells based on the NC-Nafion modified Zn electrode with different NC contents was first estimated. As shown in Fig. S14, under a current density of 0.5 mA cm^−2^ with a deposition capacity of 0.5 mAh cm^−2^, the NC-Nafion@Zn-based cell shows the longest operating time of 2598 h, indicating the corresponding content of NC is more appropriate. The reduced overpotential reflects that the NC-Nafion SEI coating stimulates the desolvation process of Zn^2+^ in the OHL and the kinetics of Zn deposition [55]. In comparison, the C-Nafion@Zn, Nafion@Zn, and bare Zn-based cells exhibit inferior lifespans of 535, 476, and 236 h at the same test condition (Fig. 3a). As the current density increased to 1 mA cm^−2^, the NC-Nafion@Zn-based cell exhibits superior cycling behavior of 3400 h, which is much better than those of C-Nafion@Zn and Nafion@Zn-based cells (1352 and 601 h, respectively), and the lifetime of NC-Nafion@Zn is about 16 times longer than that of bare Zn cells (210 h, Fig. 3b). Impressively, the NC-Nafion@Zn||NC-Nafion@Zn cell delivers an outstanding cycle life of 2000 h at 5 mA cm^−2^ and 1 mAh cm^−2^, and the charge/discharge curves in the initial and final working states demonstrate the small differences in Zn deposition overpotential (43.5 vs. 56 mV) (Fig. 3c), while the C-Nafion@Zn, Nafion@Zn, and bare Zn cells only display a short lifespan of 442, 210, and 150 h, respectively. Moreover, as the testing condition becomes harsher (10 mA cm^−2^ and 10 mAh cm^−2^), the NC-Nafion@Zn symmetric cell still achieves an attractive cycle lifespan of 200 h with a stable and low overpotential of 80 mV (Fig. S15). Whereas the other batteries present rapid failure with intense voltage polarizations (54 h for C-Nafion@Zn cell, 42 h for Nafion@Zn, 4 h for bare Zn), which may be attributed to the battery flatulence or short circuits caused by severe side reactions and dendrite growth [56]. Figure S16 displays the lifespans of different symmetrical batteries at 10 mA cm^−2^ under a Zn utilization of 57%. The NC-Nafion@Zn cell can keep stable cycling for 82 h, which is much longer than the other three cells. The excellent stability of NC-Nafion@Zn is among the best reported Zn metal anodes (Fig. 3d), and the detailed performance data are presented in Table S2 in SI. These data suggest that the NC-Nafion artificial SEI layer plays a crucial role in stabilizing the electrolyte Zn anode interface. Furthermore, the rate performance was tested in symmetric Zn||Zn cells at a series of current densities with a consistent capacity of 1 mAh cm^−2^ (Fig. 3e). As the current densities increase from 0.5 to 8 mA cm^−2^, the NC-Nafion@Zn showcases the lowest voltage hysteresis (from 25 to 83.5 mV) than those of C-Nafion@Zn and bare Zn-based cells. With that, the exchange current density (*i_0_*) was calculated using the Butler–Volmer approximation equation (Equation 1 in SI). The *i_0_* value of NC-Nafion@Zn is 6.66 mA cm^−2^, which is larger than that of C-Nafion@Zn and bare Zn (5.74 and 5.32 mA cm^−2^, Fig. 3f), suggesting that the existence of the DLC/Sn-DLC layer accelerates ion diffusion dynamics during the Zn plating/stripping process.

**Figure 4.**
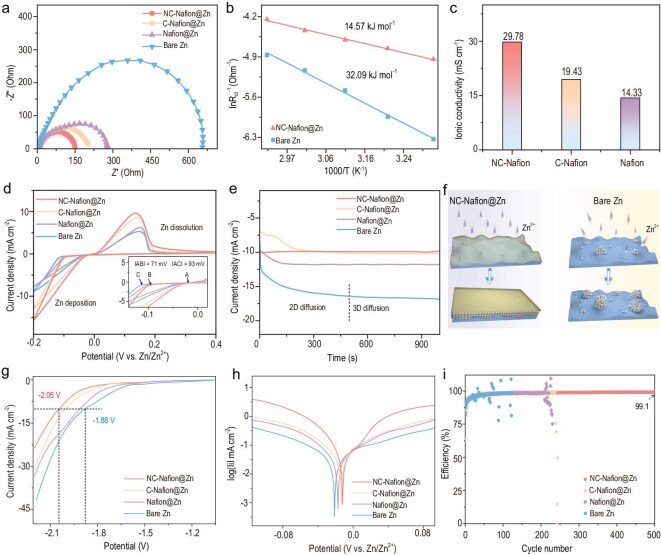
The electrochemical analysis of the NC-Nafion@Zn, C-Nafion@Zn, Nafion@Zn, and bare Zn cells: (a) Nyquist patterns; (b) Arrhenius curves; (c) ionic conductivity; (d) CV curves (inset: local magnification curves); (e) CA profiles; (f) schematic diagram of 3D and 2D diffusion models; (g) linear polarization curves in a 1 M Na_2_SO_4_ aqueous electrolyte; (h) Tafel plots; (i) CE profiles.

To demonstrate that the NC-Nafion interphase plays a prominent role in accelerating the ion transfer and dissociation process, electrochemical impedance spectroscopy (EIS) was first conducted. The Nyquist profiles in Fig. 4a represent the lowest charge transfer resistance (*R*_ct_) value of NC-Nafion@Zn based cell (150 Ω) as compared with those of C-Nafion@Zn (200.9 Ω), Nafion@Zn (277.3 Ω), and bare Zn (655.8 Ω), indicating the enhanced charge transfer kinetics. Then the desolvation energy barrier was evaluated according to the EIS profiles of different symmetrical batteries measured at a range of temperatures (30–70°C, Fig. S17). The activation energy (*E_a_*) of NC-Nafion@Zn is calculated to be 14.6 kJ mol^–1^ based on the Arrhenius Equation (Equation 2 in SI), which is smaller than half of bare Zn (32.1 kJ mol^–1^, Fig. 4b). The impaired *E_a_* value reveals NC-Nafion reduces the interaction between Zn^2+^ ions and solvent water molecules, contributing to a faster Zn^2+^ desolvation process and ion transfer kinetics [57,58]. Due to the low desolvation barrier and rational designed ionic channels, the NC-Nafion@Zn possesses a larger Zn^2+^ transference number (t_Zn_^2+^) of 0.75 than those of C-Nafion@Zn (0.56) and bare Zn (0.26) (Fig. S18). The ion conductivity of the NC-Nafion layer coated on stainless steel is computed to be 29.78 mS cm^−1^ (Fig. 4c and Fig. S19), the value is 1.53 and 2.08 times greater than those of C-Nafion (19.43 mS cm^−1^) and Nafion (14.33 mS cm^−1^), respectively. Figure 4d shows the cyclic voltammetry (CV) curves of asymmetric Zn||Ti cells assembled with different anodes. For NC-Nafion@Zn, the value of nucleation overpotential, caused by the potential difference between crossover potential and Zn^2+^ ions starting reduction, is ∼71 mV, which is smaller than the other electrodes. Meanwhile, the current density is much higher than that of the bare Zn. These results imply the accelerated ion dissolution process and transfer kinetics [59]. Moreover, the diffusion behavior of adsorbed ions at the electrode interface was estimated by the Chronoamperometry (CA) test under a constant potential of −150 mV, which plays an important role in the Zn sedimentation and growth morphology [60]. As depicted in Fig. 4e, the bare Zn electrode shows a continuous increase in current density from −8.5 to −16.4 mA cm^–2^ beyond 500 s, indicating that the deposition of Zn^2+^ is dominated by a long and rampant 2D diffusion behavior, due to the sluggish Zn^2+^ ions diffusion process and vertical deposition (Fig. 4f), which produces unfavorable Zn dendrite growth [61]. The C-Nafion@Zn and Nafion@Zn achieve stable current density after ∼260 and 190 s, then display a stable 3D diffusion process. Impressively, a small and stationary current density is observed on the NC-Nafion@Zn electrode, the stable 3D diffusion process suggests that the NC-Nafion artificial SEI layer can constrain the nucleation of Zn^2+^, forming a flat and homogeneous zinc deposition layer [62]. In addition, the enhancement interfacial stability of NC-Nafion@Zn was analyzed by hydrogen evolution polarization curves and Tafel plots. Under the same current density (10 mA cm^–2^), the HER potential of NC-Nafion@Zn based symmetric cell is −2.05 V (Fig. 4g), the value is much lower than those of C-Nafion@Zn (−2.0 V), Nafion@Zn (−1.91 V), and bare Zn (−1.88 V). The Tafel plots further evidence the lower corrosion current and higher corrosion voltage of NC-Nafion@Zn than the other electrodes (Fig. 4h). The results indicate that the NC-Nafion endows the Zn foil with HER activity inhibition and improved anti-corrosion ability [63], thus resulting in effective suppression of electrode surface corrosion and side reactions induced by water decomposition. As a consequence, the CE of asymmetric NC-Nafion@Zn||Cu cell exhibits a stable and high average CE of 99.1% over 500 cycles at 0.5 mA cm^−2^ and 0.5 mAh cm^−2^ (Fig. 4i), while the CE of the C-Nafion@Zn, Nafion@Zn, and bare Zn are undulant and deliver a cyclic lifetime of only 240, 230, and 124 cycles, respectively, evidencing the improved Zn plating/stripping reversibility. These experimental results further validate that the NC-Nafion artificial SEI layer, relying on the synergistic effect of Nafion and high Fermi-level of NC, not only achieves the sieving and capture of Zn^2+^ ions and accelerates their transport kinetics, but also reduces the dissociation barrier and enhances interface stability.

**Figure 5.**
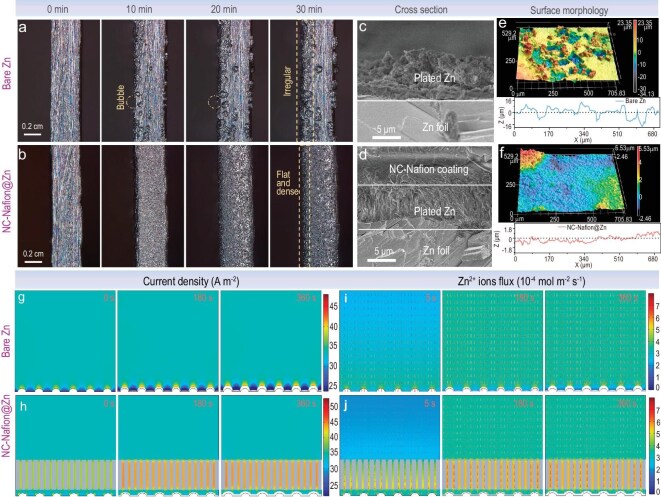
The morphology evolvement of Zn ions on different substrates: *in-situ* super-depth microscopy images of Zn deposition on (a) bare Zn and (b) NC-Nafion@Zn electrodes at a current density of 10 mA cm^–1^; cross-sectional SEM and surface LSCM images and corresponding surface roughness curve of (c, d) bare Zn and (e, f) NC-Nafion@Zn anodes. The COMSOL evolution simulation of (g, h) current density distribution, (i, j) Zn^2+^ ions flux distribution for the bare Zn and NC-Nafion@Zn anodes.

The surface morphology and crystalline phase evolution of Zn ion deposition was characterized by *in-situ* observations using super-depth microscopy, SEM, laser scanning confocal microscopy (LSCM), and XRD measurements. As shown in Fig. 5a and Video 4, during 30 mins of continuous Zn^2+^ ions plating in 2 M ZnSO_4_ aqueous electrolyte under a large current density of 10 mA cm^–1^, plenty of protrusions are observed after working for 10 min and continue to grow on the surface of the bare Zn electrode, indicating uncontrolled growth of Zn dendrites, which will further exacerbate the inhomogeneous electric distribution and Zn^2+^ ions flux [64]. In sharp contrast, the NC-Nafion@Zn electrode continuously maintains a dense and flat intersecting surface without any dendrites (Fig. 5b, Video 5). Moreover, the cross-sectional and surface morphologies of different electrodes after 20 cycles at a current density of 1.0 mA cm^–2^ and a capacity of 1.0 mAh cm^–2^. From the cross-sectional view SEM in Fig. 5c, it can be observed that the plated Zn on the bare Zn anode is loose and rough, meanwhile, many chaotic and vertical Zn dendrites grow on its surface (Fig. S20). However, the NC-Nafion@Zn retains a smooth and dendrite-free surface morphology, which is similar to the initial state (Fig. S21). The related sectional drawing in Fig. 5d evidenced that the densely plated Zn is homogeneously and closely located between the NC-Nafion interfacial layer and Zn substrate without obvious gaps. Furthermore, the uneven surface and the production of by-products were further validated by 3D LSCM images and XRD profile. Numerous protrusions and indentations appeared on the bare Zn electrode with a considerable surface height difference of 31.41 μm (Fig. 5e), and the corresponding XRD in Fig. S22 confirms that the peaks appearing at 12.3, 16.1, and 24.5° belonged to the ZSH (PDF: 39–0690), indicating the formation of uncontrolled by-products. While the NC-Nafion@Zn displays a flat surface with a small height difference of 2.48 μm (Fig. 5f), there was a negligible ZSH-related peak in its XRD curve, thus the collaboration of NC and Nafion effectively inhibited the side reactions. These results demonstrate that NC-Nafion can act as a functional artificial SEI layer to modulate uniform deposition of Zn^2+^ ions and restrain interface side reactions.

To deeply understand the modulating mechanism of the NC-Nafion interface layer on Zn^2+^ deposition behavior, a COMSOL Multiphysics simulation was conducted based on finite element modeling (FEM) to visualize the dynamic evolution differences of the electric field and Zn^2+^ ions flux on bare Zn and NC-Nafion@Zn electrodes. The initial model of bare Zn and NC-Nafion@Zn electrodes is shown in Fig. S23. After implementing a current density of 5 mA cm^–2^, inhomogeneous current density and Zn^2+^ ion flux distributions are observed on the bare Zn surface (Fig. 5g, h), which especially accumulate on the Zn protrusions to form Zn deposition ‘hot spots’ and produce Zn dendrites. This process will further exacerbate the distribution differences of current density and Zn^2+^ ion flux in the tips and valley regions, which induce uncontrollable dendrite growth during the electrodeposition process. On the contrary, the NC-Nafion@Zn electrode in Fig. 5i and j shows homogeneous and enhanced current density and Zn^2+^ ion flux throughout the entire Zn deposition operation, and the deposited Zn is uniformly distributed under the NC-Nafion coating, which is consistent with the observed morphology in the experiment. Moreover, their corresponding continuous evolution of current density and Zn^2+^ ion flux within 360 s are recorded in the picture of GIF 1–4 in the online Supplementary File.

**Figure 6.**
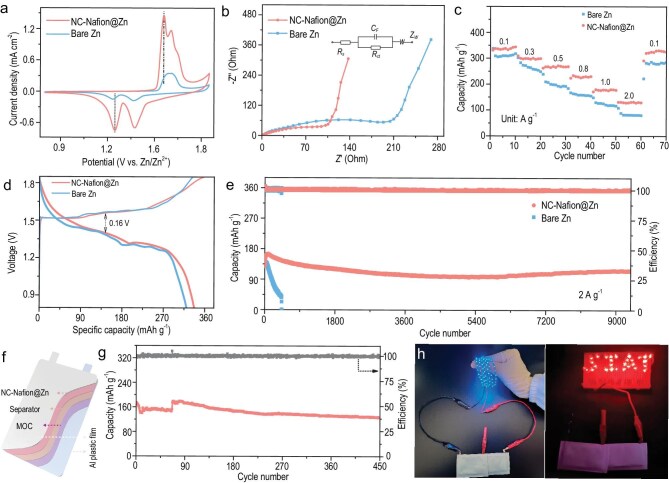
Electrochemical performance of full cells. (a) CV curves. (b) The fitting Nyquist plots based on the insert equivalent circuit model. (c) Rate performances. (d) The typical voltage profile at 0.1 A g^–1^. (e) The long-term cycling stability and corresponding CE at 2.0 A g^–1^. (f) Schematic diagram of NC-Nafion@Zn||MOC pouch batteries. (g) Cycling properties of NC-Nafion@Zn||MOC cells at 1.0 A g^−^^1^. (h) Photographs of two tandem pouch batteries lighting up the LED light array.

To illustrate the feasibility and practicality of NC-Nafion@Zn, the performances were estimated using the coin and pouch full battery assembled with the composite of manganic manganous oxide (Mn_3_O_4_) nanoparticles and carbon nanotubes (CNTs) cathode (named as MOC). The XRD and SEM images indicate that pure-phase Mn_3_O_4_ nanoparticles with a size of 30–50 nm were uniformly adhered to the interpenetrating CNT networks (Figs S24 and S25). The CV curves in Fig. 6a reveal that the NC-Nafion@Zn||MOC battery shows a smaller polarization voltage and larger current density, demonstrating its reduced electrochemical polarization and higher capacity [65]. The fitting Nyquist profile (Fig. 6b) of the NC-Nafion@Zn anode displays an obviously smaller *R_ct_* value than that of the bare Zn anode (91.4 vs. 198.9 Ω, Table S3), implying a faster Zn^2+^ ion reaction kinetics. Then the rate performances were conducted at current densities of 0.1 to 2.0 A g^–1^. As shown in Fig. 6c, the NC-Nafion@Zn||MOC battery provides higher specific capacities of 337.6, 298.8, 270.1, 230.3, 177.8, and 130.1 mAh g^–1^ and exhibits a rapid response as the current density recovers. The homologous charge/discharge curves at each current density are shown in Fig. S26. Typically, the charge/discharge curves at 0.1 A g^–1^ illustrate that NC-Nafion@Zn possesses a narrow voltage difference and longer voltage plateaus (Fig. 6d). In addition, the NC-Nafion@Zn-based battery achieves an outstanding long-term cycling stability of 9300 cycles at current densities of 2.0 A g^–1^ (Fig. 6e), The specific capacity is 116.7 mAh g^–1^ and maintains a high-capacity retention of 91.3%. The cycling performance is very competitive compared to that of the best reported full battery (Table S4). In sharp contrast, the bare Zn based battery displays rapid capacity decay and short cycle life. After 430 cycles, the capacity retention is only 28.5%. These results reflect that the NC-Nafion artificial SEI layer effectively suppresses Zn dendrites and side reactions during battery operation, improving the cycling lifespan and rate performance. Moreover, a pouch cell of NC-Nafion@Zn||MOC was fabricated to provide further proof-of-concept (Fig. 6f). It delivers an excellent reversible capacity of 127.2 mAh g^–1^ after 450 cycles with a retention of 71.7% (Fig. 6g). Two connected pouch batteries can power the light-emitting diodes (LEDs) light array (a total of 137 blue LED monomers are included, Fig. 6h), demonstrating the prospective application foreground.

## CONCLUSION

In summary, we developed a feasible NC-Nafion artificial SEI layer integrating broad ion sieving channels and Fermi-level engineering. The NC-Nafion artificial SEI layer can selectively block harmful anions and increase Zn^2+^ flux through electrostatic interactions. The high Fermi-level NC in NC-Nafion activates the dehydration of [Zn(H_2_O)_6_]^2+^ in the OHL through a non-coordinating interaction, which eliminates the solvated H_2_O-induced side reactions and promotes charge transfer dynamics. As a result, the NC-Nafion@Zn symmetric cells achieve a high CE of plating/stripping and deliver an excellent lifespan of 3400 h with a low overpotential under a current density of 1 mA cm^−2^. The NC-Nafion@Zn//Mn_4_O_3_-CNTs full battery shows an impressively long lifespan of 9300 cycles at 2 A g^–1^, accompanied by a high capacity of 116.7 mAh g^–1^ and a retention of 91.3%. This work provides a new perspective for the interphase design with Fermi-level engineering and a non-coordinated charge transfer process.

## Supplementary Material

nwaf070_Supplemental_Files
