# Supplementary material for: Non-coordinating charge transfer enables ultrafast desolvation of hydrated zinc ions in the outer Helmholtz layer for stable aqueous Zn metal batteries
Source: Natl Sci Rev. 2025 Feb 22;12(4):nwaf070. doi: 10.1093/nsr/nwaf070 (PMC11960093; doi:10.1093/nsr/nwaf070)
Supplement: nwaf070_Supplemental_Files [file nwaf070_supplemental_files.zip › Supplementary data.pdf]

Supporting Information

Non-coordinating charge transfer enables ultrafast desolvation of hydrated zinc ions in the outer Helmholtz layer for stable aqueous Zn metal batteries

Xiuli Guo[1†], Qiaoling Peng[1,2†], Rui Yang[1†], Gengyou Cao[1,2], Jianfeng Wen[1,3], Kyungsoo Shin[1,3], Ye Zheng[1,2], Sarayut Tunmee[4], Caineng Zou[5*], Yongping Zheng[1,3*], Xiaolong Zhou[1,3*], Yongbing Tang[1,3*]

1. Advanced Energy Storage Technology Research Center, Shenzhen Institute of Advanced Technology, Chinese Academy of Sciences, Shenzhen, 518055, China
2. Nano Science and Technology Institute, University of Science and Technology of China, Suzhou 215123, China
3. Shenzhen College of Advanced Technology, University of Chinese Academy of Sciences, Shenzhen, 518055, China
4. Synchrotron Light Research Institute (Public Organization), 111 University Avenue, Muang District, Nakhon Ratchasima 30000, Thailand
5. PetroChina Shenzhen New Energy Research Institute

Email: zcn@petrochina.com.cn (C.N. Zou); yp.zheng@siat.ac.cn (Y.P. Zheng); zhouxl@siat.ac.cn (X.L. Zhou); tangyb@siat.ac.cn (Y.B. Tang)

†X.L. Guo, Q.L. Peng, and R. Yang contributed equally to this work.

Key words: Fermi-level engineering, non-coordinating charge transfer, fast desolvation, nitrogen-doped amorphous carbon, aqueous Zn metal batteries

1. Experimental section

Materials

Citric acid monohydrate ($C_6H_8O_7{\cdot}H_2O$, AR, 99%), urea ($CO(NH_2)_2$, AR, 99%), ethanol (C_2H_5OH, AR, 99%), N,N-Dimethylformamide (DMF, AR, 99%) and $Mn(CHCOO)_2{\cdot}4H_2O$ (AR, 99%) were provided by Aladdin Industrial Co., Ltd. (Shanghai, China). Nafion solution (D520, 5 wt.%) was purchased from DuPont. Zinc (Zn) foil (thickness of 50 μm), titanium (Ti) foil (thickness of 10 μm), copper (Cu) foil (thickness of 20 μm) and poly(vinylidene fluoride) (PVDF), conductive carbon black (Super P) were provided by Shenzhen Kejingstar Technology Ltd. (China). Carbon nanotubes (CNTs) and glass microfiber separators (GF/A) were obtained from XFNANO and Whatman. All chemicals were used without further purification.

Preparation of nitrogen-doped amorphous carbon nano-sheet (NC)

NC was prepared through mixing under heating conditions, solvent evaporation, and pyrolysis process.[1] First, 1.0 g of $C_6H_8O_7{\cdot}H_2O$ and 10.0 g of $CO(NH_2)_2$ were dissolved into 200 mL of deionized water and ethanol (3:1, v:v), and then stirred in an oil bath at 75°C for 5 h. Next, the obtained mixture was kept at 100°C. After the solvent was completely evaporated, the precursor was heated for 2 h at 350 and 600°C in an argon atmosphere, respectively, with corresponding heating rates of 2 and 5 °C min^{-1}, finally, the NC powders were achieved.

Preparation of NC-Nafion@Zn, C-Nafion@Zn, and Nafion@Zn electrodes

4 mL of Nafion liquid, 8 mL of DMF, and 15 mg of NC powders were added into a 20 mL glass bottle, then stirred and sonicate for 1 h each time, repeating four times

alternately to obtain a homogeneous solution. The above solution was poured into a groove of 7*7*1 cm^3 made of polished commercial zinc foil, and placed it in a vacuum drying oven at 80°C for 24 h. The decorated zinc foil was immersed in 2 M $ZnSO_4$ solution for 24 h to sufficient ion-exchange and remove the H^+ in Nafion. Then wash them thoroughly with deionized water and dry them in room in a vacuum drying oven at 60°C to obtain NC-Nafion@Zn sample. For comparison, a series of NC-Nafion interphases with different NC contents were prepared through the above processes. As the added mass of NC is 5, 10, and 20 mg, name the electrodes as NC-Nafion@Zn-1, NC-Nafion@Zn-2, and NC-Nafion@Zn-3, respectively.

The fabrication process of pure carbon (C) modified Nafion anodes (C-Nafion@Zn) and Nafion@Zn anode are similar to that of NC-Nafion@Zn, except replacing NC with C, or no additives.

Preparation of Mn_3O_4 nanoparticles and CNT complex (MOC) cathode

Manganic manganous oxide (Mn_3O_4) nanoparticles composite with CNTs cathode powders (named MOCs) were synthesized according to the reported literature.[2] In brief, solution A is an ethanol solution (10 mL) containing 80 mg of CNTs, solution B is an ethanol solution (20 mL) containing 1.0 g of $Mn(CHCOO)_2 \cdot 2H_2O$. Then the solution B was slowly added into solution A under continuous stirring state for 30 min, and the resulting mixture was transferred into a 50 mL Teflon-lined autoclave and heated at 160°C for 8 h. Finally, the powders were collected and pyrolyzed at 300°C for 2 h with a heating rate of 3 °C min^{-1} in a muffle furnace.

The MOC cathode was prepared by mixing MOC active material, Super P, and PVDF

in NMP solvent with a mass ratio of 7:2:1, and then evenly coated on the Ti foil. After fully drying in a vacuum oven at 80℃, the MOC cathode is achieved. The load of active substances is controlled between 1.0–2.0 mg cm^{-2}.

Fabrication of batteries and electrochemical measurement

For the asymmetric Zn||Ti, Zn||Cu, and symmetric Zn||Zn cells, a piece of a round disk of Zn foil (working electrode) was paired with Ti, Cu or Zn foil counter electrodes, separately. The diameters of all electrodes are 10 mm. 2 M $ZnSO_4$ (80 μL) was used as electrolyte. A glass fiber (diameter of 16 mm) was used as the separator. The full CR2032 cell of Zn||MOC was fabricated by the prepared MOC cathode, Zn foil anode, glass fiber separator, and 2 M $ZnSO_4$ + 0.2 M $MnSO_4$ of aqueous electrolyte solution. The pouch batteries used the same electrode materials and electrolyte as the coin cells, and the size of the cathode and NC-Nafion@Zn anode were controlled to be 2x2 cm^{-2} with an N/P ratio of approximately 80.8.

Material characterization

The evolution of morphology and microstructures and compositions of all obtained samples were characterized by scanning electron microscopy (SEM, Hitachi SU-8020 and Hitachi, S-4800) and transmission electron microscopy (TEM, FEI Tecnai JEM-F200) with energy-dispersive spectroscopy (EDS), laser scanning confocal microscope (CLSM, KEYENCE, VK-X1100), ultra depth of field digital microscope (Leica DVM6 M), X-ray diffraction (XRD, Rigaku MiniFlex 600 X-ray, Cu *K*α radiation, λ=1.54 Å), X-ray photoelectron spectroscopy (XPS, Thermo SCIENTIFIC Nexsa), Raman spectra (HORIBA XpoRA PULS, excitation wavelength: 532 nm). The cross-sectional images

were observed by fracturing the samples in liquid nitrogen. The zinc affinity was tested by contact angle (CA) measurement (POWEREACH, SCI6000E). The content of N element was obtained by organic elemental analysis (Elementar Vario EL). CV, CA, EIS, LSV, and Tafel curves were tested by Princeton electrochemical workstation (Versa-STAT 3F). The cycling performances of CR2032-type coin cells, including Zn||Cu, Zn||Zn, and Zn||MOC were measured by an NEWARE CT-4008 equipment.

To estimate the Zn^{2+} diffusion dynamics during Zn platting/stripping process, the exchange current density (i_0) was calculated based on the Butler–Volmer approximation equation:

$$i = i_0 \frac{\eta F}{2RT} \qquad \textbf{(Equation 1)}$$

where i and η are the applied current density and overpotential corresponding to the rate test, F is the Faraday constant, R is the ideal gas constant, and T is the Kelvin temperature.

The activation energy (E_a) is calculated by the following Arrhenius equation to evaluate the Zn^{2+} ions desolvation process.

$$\frac{1}{R_{ct}} = \mathrm{A}\exp\left(\frac{-E_a}{\mathrm{R}T}\right) \qquad \textbf{(Equation 2)}$$

where A is the frequency factor, R is the ideal gas constant, T is the absolute temperature, and R_{ct} is the charge transfer resistance.

The Zn^{2+} ions transference numbers ($t_{Zn^{2+}}$) are determined by combining the EIS values of the Zn||Zn battery before and after the CA test program and calculated based on the following equation:

$$t_{Zn^{2+}} = \frac{I_S(\Delta V - I_0 R_0)}{I_0(\Delta V - I_S R_S)} \qquad \textbf{(Equation 3)}$$

where ΔV is the voltage polarization applied (30 mV), I_S and R_S are the steady state current and resistance, respectively, and I_0 and R_0 are the initial current and resistance, respectively.

The ionic conductivity of NC-Nafion and Nafion layers was estimated by two blocking electrodes (Ti foil) and calculated by the following equation:

$$\sigma = \frac{L}{RS} \qquad \textbf{(Equation 4)}$$

where R is the resistance according to EIS measurement, L represents the thickness of the NC-Nafion and Nafion coatings, and S is the area of the contact between Ti foil and the electrolyte (0.785 cm^2).

The water uptake of NC-Nafion and Nafion films is quantified by swelling rate (SR) according to the following formula:

$$SR = \frac{\mathrm{m}_{wet} - \mathrm{m}_{dry}}{\mathrm{m}_{dry}} \times 100\% \qquad \textbf{(Equation 5)}$$

where m_{wet} represent the mass of samples that fully swell after being immersed in deionized water for 48 h, and the surface water is absorbed using filter paper. m_{dry} is the samples' mass under the completely dried state.

2. Calculation methods

Theoretical calculations were based on density functional theory (DFT),[3, 4] and performed with the Vienna Ab initio simulation package.[5] For the exchange-correlation interaction, the generalized gradient approximation employing the Perde-Burke-Ernzerhof function was performed.[6] The cutoff energy of the projector-augmented wave potential was set at 450 eV for structural relaxation.[7] When the difference in energy between two steps was less than 1.0×10^{-5} eV, the electronic

relaxation loops terminated. Because the hydrated zinc ions are charged, structures of the hydrated zinc ions and hydrated zinc ions adsorbed on carbon/N-carbon were optimized in a 30*30*30 Å cubic cell. The cell structures were fixed during relaxation and the Monkhorst-Pack k-mesh was set up as 1*1*1. In $[Zn(H_2O)_6]^{2+}$, the distances between Zn and O are around 2.12 Å. When the distance between Zn and O increases to 2.5 Å during the subsequent relaxation process, it is defined as the desolvation of hydrated zinc ions by the removal of water molecules. The adsorption energy (E_{ad}) between carbon/N-carbon and hydrated zinc ion is defined as follows:

$$E_{ad} = E_{complex} - E_c - E_{Zn} \qquad \textbf{(Equation 6)}$$

where E_{Complex} is the total energy of the hydrated zinc ion adsorbed on carbon/N-carbon complex, E_{Zn} is the energy of the hydrated zinc ion, and E_{C} is the energy of pure C and NC.

COMSOL Simulation Method

Utilizing COMSOL Multiphysics simulation software, a fully coupled transient simulation of an ion battery model was conducted, integrating electrochemistry with a deformable mesh. The simulation began with the initialization of boundary potential, electrode kinetics, and boundary geometry distribution of the ion battery. Electrode kinetics were modeled based on the Butler-Volmer expression, and electrolyte transfer adhered to the Bruggeman rule. The anode and both lateral boundaries were maintained as undeformed, whereas the cathode boundary was modeled as a deformable electrode surface, coupled with the electrochemical interface of the ion battery module. The electrochemical process simulation was conducted within a 50*50 μm area. The initial shape of the bare Zn foil at the lower boundary was configured as a semi-elliptical array.

Its surface was set as a deformable boundary. Parameters corresponding to different materials and pore environments were established, with the anode and cathode set at transmission coefficients of 1 and 0 V, respectively, to ensure consistency in the initial growth interface. Adjustments to various material environments were achieved by specifying the ions involved in the reaction, adjusting the exchange current, modifying growth boundary parameters, and altering the degree of electron participation in the chemical reactions. This simulation enabled the derivation of the initial state and pore state, along with distributions of ion flux, electrolyte current density, and electrode current density within the framework of electrochemical reactions. Additionally, the NC Nafion interface layer was designed as a rectangular array with a width of 2 μm and a spacing of 1 μm.

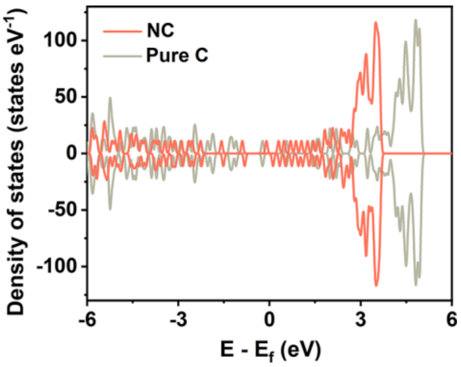

Figure S1. The density of states for NC and pure C.

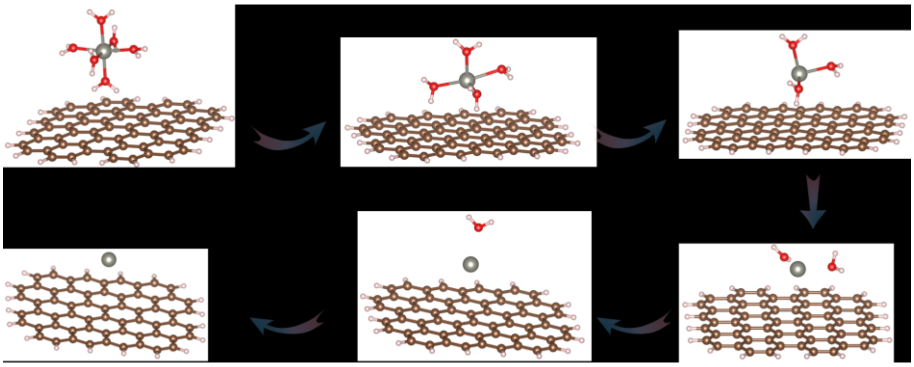

Figure S2. The desolvation evolution process of $Zn(H_2O)_6^{2+}$ on pure C.

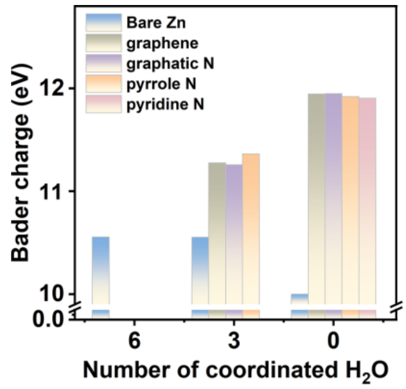

Figure S3. The barde charge evolution of Zn^{2+} on different substrates.

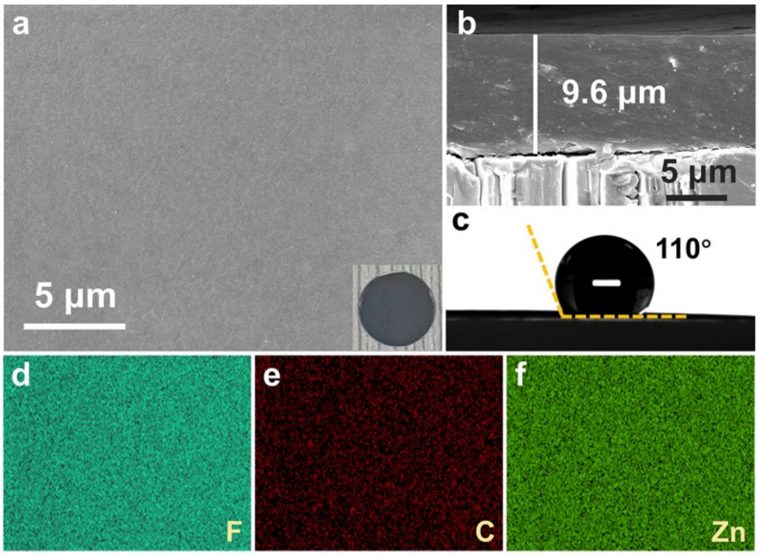

Figure S4. (a) SEM image, (b) cross-sectional SEM image of C-Nafion@Zn. (c) Contact angle, and (d–f) EDS mapping of F, C, and Zn elements of C-Nafion@Zn. Insert shows the corresponding optical picture of the C-Nafion@Zn anode.

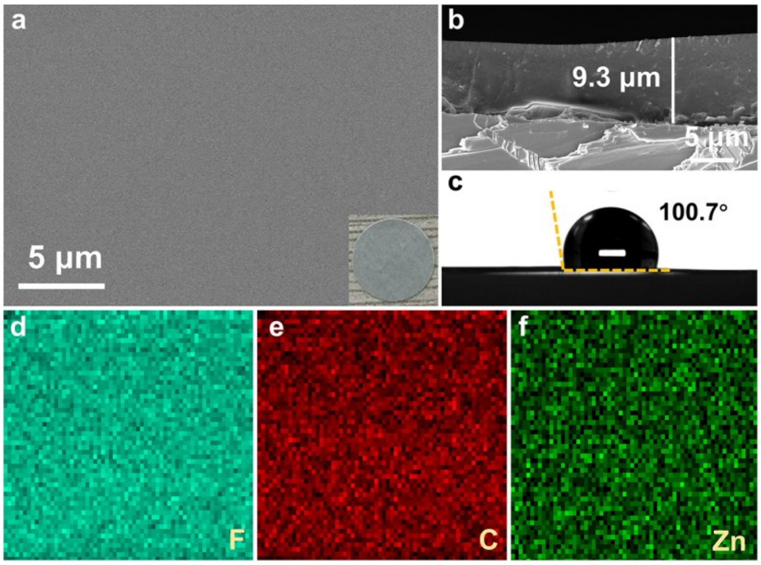

Figure S5. (a) SEM image, (b) Cross-sectional SEM image of Nafion@Zn. (c) Contact angle, and (d–f) EDS mapping of F, C, and Zn elements of Nafion@Zn sample. Insert shows the corresponding optical picture of the Nafion@Zn anode.

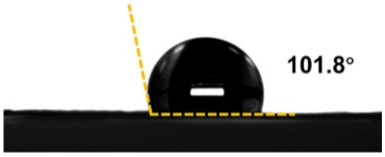

Figure S6. Contact angle of bare Zn anode.

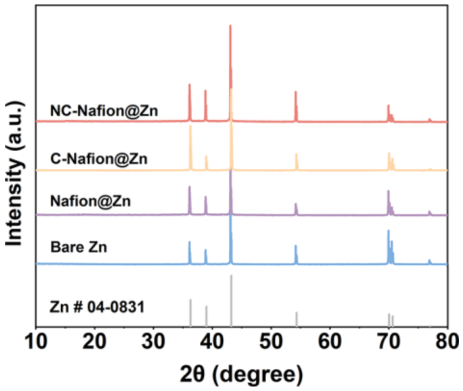

Figure S7. The XRD profiles of different electrodes.

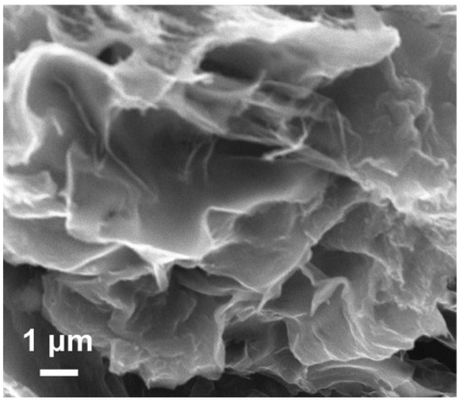

Figure S8. The SEM image of pure carbon material.

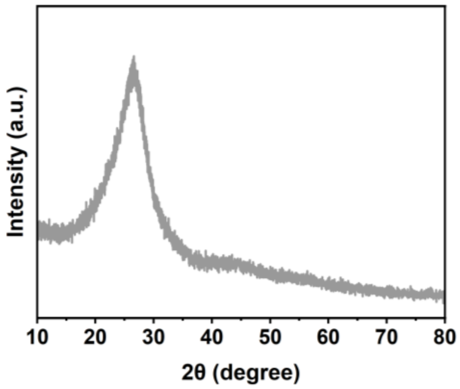

Figure S9. The XRD curve of NC powders.

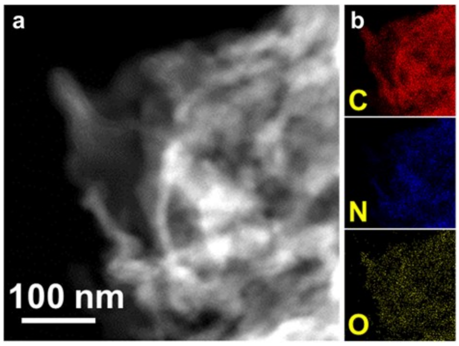

Figure S10. The HAADF image and EDS mappings of NC.

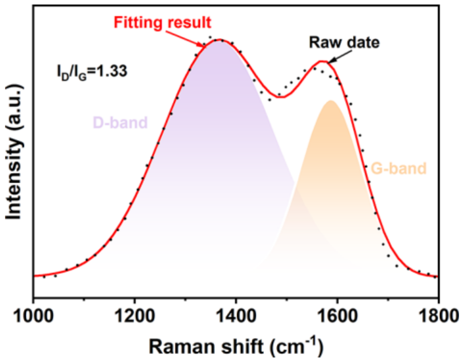

Figure S11. Raman spectrum of NC.

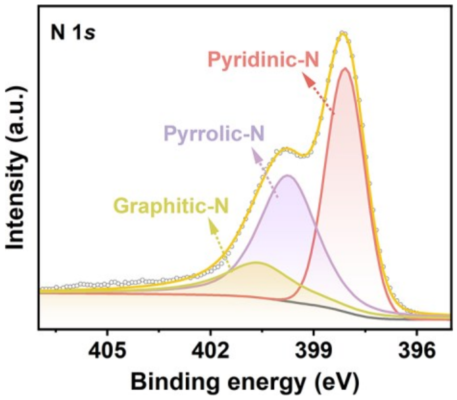

Figure S12. N 1*s* XPS spectrum of NC.

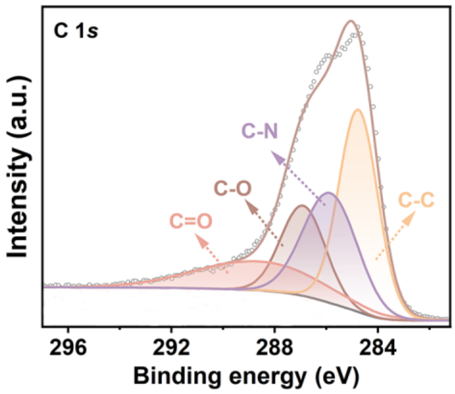

Figure S13. C 1*s* XPS spectrum of NC.

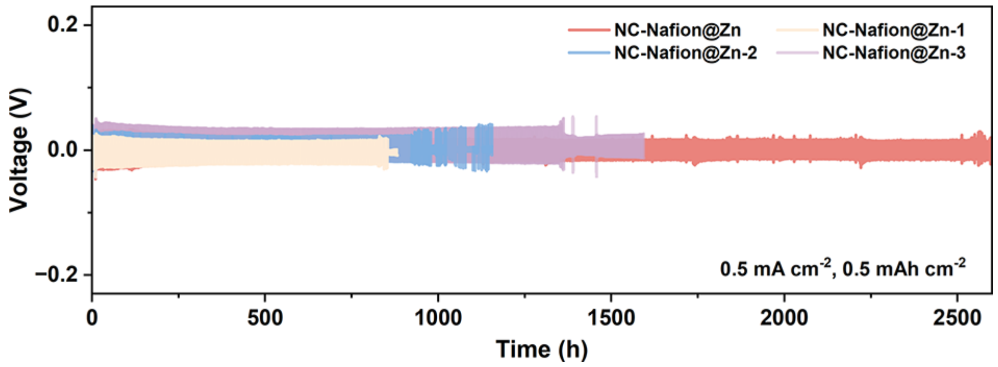

Figure S14. Discharge and charge voltage profiles of NC-Nafion@Zn-based symmetric cells with different contents of NC (that the added mass of NC in the Nifion is 5, 10, 15, and 20 mg, named the electrodes as NC-Nafion@Zn-1, NC-Nafion@Zn-2, NC-Nafion@Zn, and NC-Nafion@Zn-3, respectively) at 0.5 mA cm^{-2} and 0.5 mAh cm^{-2}.

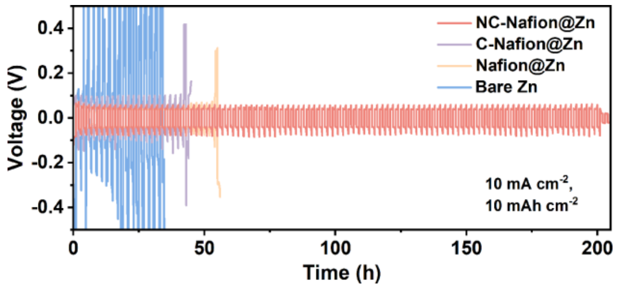

Figure S15. Long-term cycling performance of the NC-Nafion@Zn, C-Nafion@Zn, Nafion@Zn, and bare Zn symmetric cells at 10 mA cm^{-2} and 10 mAh cm^{-2}.

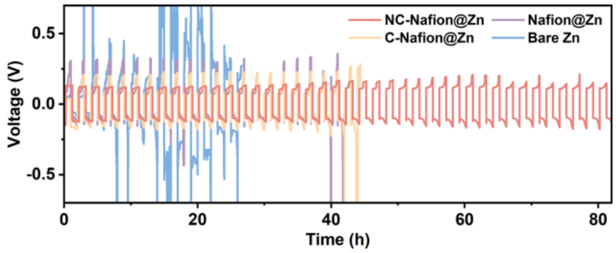

Figure S16. The cycling performance of the NC-Nafion@Zn, C-Nafion@Zn, Nafion@Zn, and bare Zn cells under a Zn utilization of 57% at 10 mA cm^{-2}.

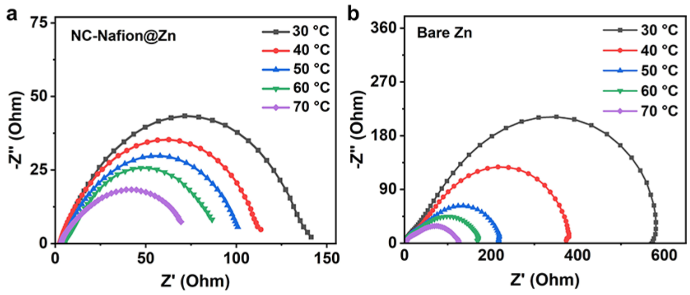

Figure S17. The Nyquist plots of the symmetric cells assembled with NC-Nafion@Zn (a) and bare Zn (b) electrodes at various temperatures.

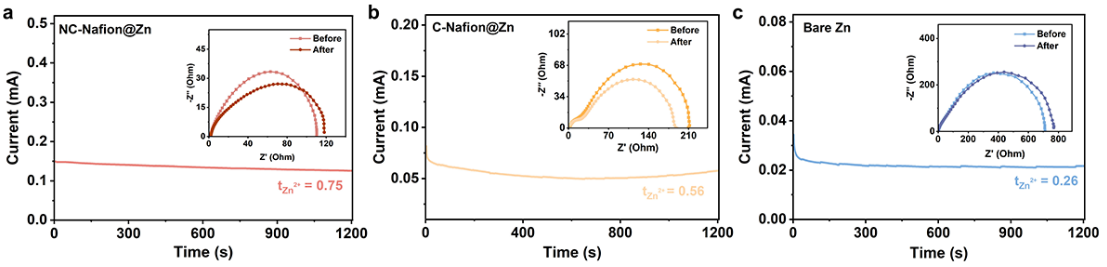

Figure S18. Current–time curves of NC-Nafion@Zn (a), C-Nafion@Zn (b), and bare Zn (c) based symmetric cells at a constant potential of 30 mV. Inset: the Nyquist plots of the symmetric cells before and after the CA test.

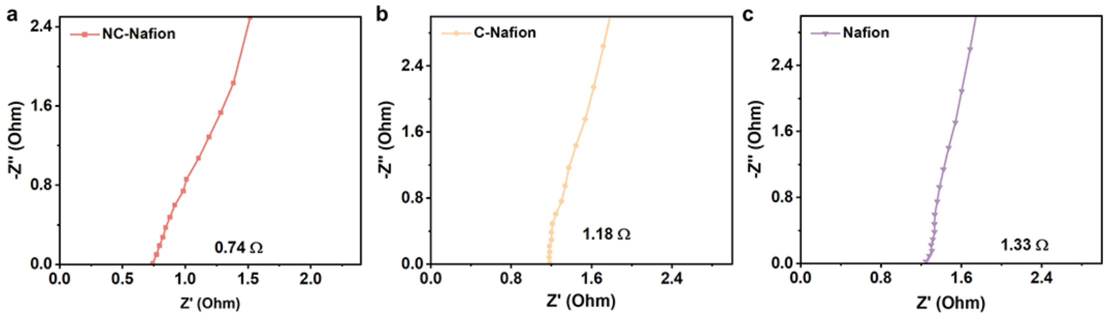

Figure S19. The Nyquist plots for the symmetric cells assembled with NC-Nafion (a), C-Nafion (b), and Nafion (c) layer coated on stainless steel electrodes.

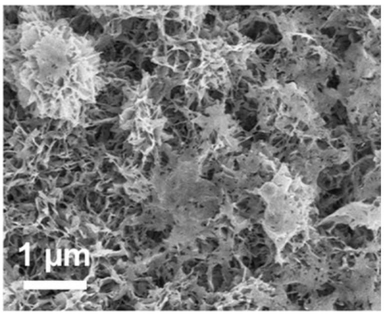

Figure S20. SEM image of bare Zn after 20 cycles (1.0 mA cm^{-2} and 1.0 mAh cm^{-2}).

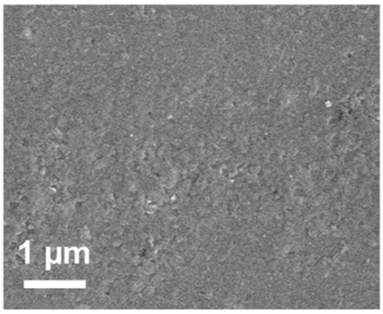

Figure S21. SEM image of NC-Nafion@Zn after 20 cycles (1.0 mA cm^{-2} and 1.0 mAh cm^{-2}).

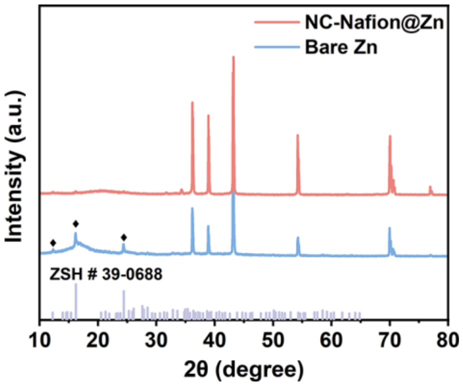

Figure S22. XRD patterns of the Zn-deposited NC-Nafion@Zn and bare Zn electrodes after 20 cycles with a capacity of 1.0 mAh cm^{-2} at 1.0 mA cm^{-2}.

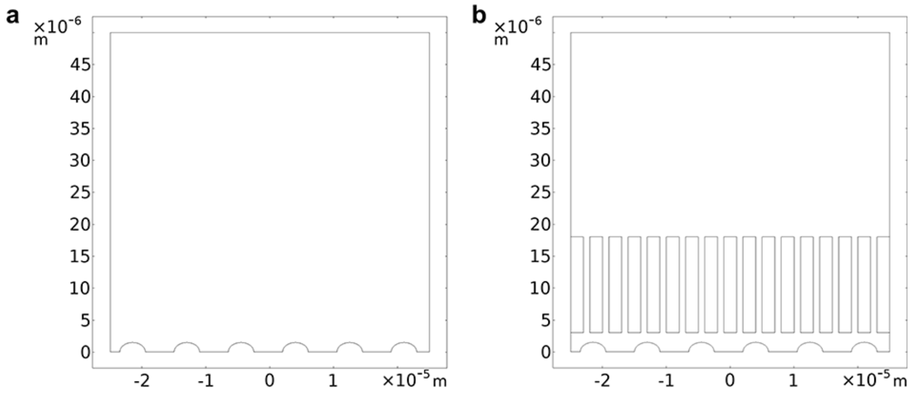

Figuer S23. COMSOL simulation initial model of bare Zn (a) and NC-nafion@Zn (b) electrode.

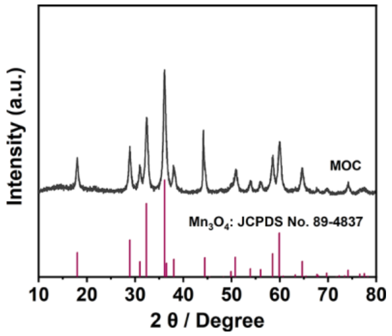

Figure S24. XRD profile of the MOC powders.

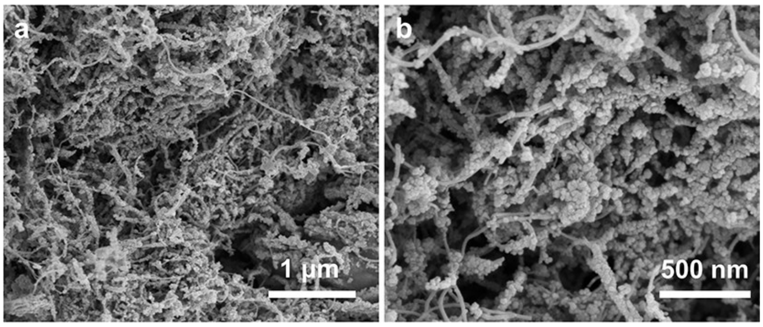

Figure S25 SEM images of MOC powders with different magnifications.

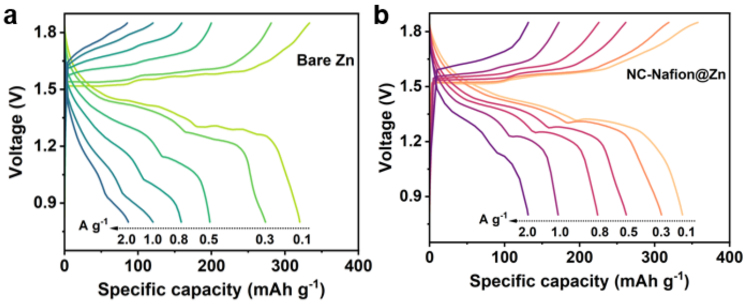

Figure S26. Charge/discharge plots of NC-Nafion@Zn (a) and bare Zn (b).

Table S1. The specific dissociation energies of $[Zn(H_2O)_6]^{2+}$ on different substrates.

	**graphitic N**	**pyridinic N**	**pyrrolic N**	**graphene**
$[Zn(H_2O)_5]^{2+}$	-0.17596	-0.09487	-0.21566	-0.10952
$[Zn(H_2O)_4]^{2+}$	-0.17596	-0.12621	-0.21864	0.85605
$[Zn(H_2O)_3]^{2+}$	-0.17596	-0.16635	-0.24307	1.28099
$[Zn(H_2O)_2]^{2+}$	-0.59845	-0.40496	-0.42696	1.82974
$[Zn(H_2O)]^{2+}$	-0.59845	-0.52179	-0.43065	1.94637
Zn^{2+}	-0.59845	-0.68702	-0.49372	2.09424

Table S2. Cycle performance comparison of NC-Nafion@Zn and some reported anodes.

**Zn anode**	**Current density** (mA cm^{-2})	**Areal capacity** **(**mAh cm^{-2}**)**	**Lifespan (h)**	**Ref**
**NC-Nafion@Zn**	**0.5**	**0.5**	**2598**	**This work**
	**1**	**0.5**	**3400**	
	**5**	**1**	**2008**	
HMCSST-Zn	3	3	1300	*Adv. Energy Mater.* **2024**, *14*, 2304138.[8]
PVDF-Sn@Zn	1	1	1200	*Nat. Commun.* **2023**, *14*, 641.[9]
Porous Cu@Zn	1	3	200	*Adv. Mater.* **2022**, *34*, 2200782.[10]
Reed interlayer	3	0.5	1455	*Adv. Energy Mater.* **2024**, *14*, 2400033.[11]
FAG@Zn	1	0.5	4000	*ACS Nano* **2023**, *17*, 21893.[12]
Zn@ZSO	1	0.25	2500	*Matter* **2022**, *5*, 4363.[13]
PI-DT-COF@Zn	5	1	500	*Angew. Chem. Int. Ed.* **2024**, e202403918.[14]
MXene10ˆZn anode	5	1	650	*Adv. Energy Mater.* **2024**, 2400318.[15]
KL-Zn	4.4	1.1	800	*Adv. Funct. Mater.* **2020**,

				30, 2000599.[16]
Zn-Sn-Bi@Zn	1	0.25	3000	*Adv. Funct. Mater.* **2024**, 2403222.[17]
ZF@F-TiO_2	1	1	460	*Nat. Commun.* **2020**, *11*, 3961.[18]
ZnS@Zn-350	2	2	1100	*Adv. Mater.* **2020**, *32*, 2003021.[19]
PVB@Zn-PVB@Zn	0.5	0.5	2200	*Adv. Funct. Mater.* **2020**, *30*, 2001263.[20]
NFZP@Zn	0.5	0.25	2800	*Energy Storage Mater.* **2022**, *47*, 491.[21]
NFSS@Zn	1	1	1500	*Adv. Sci.* **2023**, *10*, 2303343.[22]
Zn@Nafion-Zn-X	10	1	1000	*Angew. Chem. Int. Ed.* 2020, *59*, 16594.[23]
MOF-E@Zn	2	2	900	*Angew. Chem. Int. Ed.* **2023**, *62*, e202304503.[24]

Table S3. The fitting results of EIS plots of NC-Nafion@Zn//MOC and bare Zn//MOC batteries.

**Battery**	R_s	R_{ct}
NC-Nafion@Zn//MOC	0.7	91.4
Bare Zn//MOC	2.9	198.9

Table S4. Comparison of long cycling performances between the designed NC-Nafion@Zn||MOC full battery and other reported work.

Electrode materials	Current density (mA g^{-1})	Cycle numbers	Specific capacity (mA h g^{-1})	capacity retention (%)	Ref
**NC-Nafion@Zn||MOC**	**2000**	**9300**	**116.7**	**91.3**	**This work**
MnO_2@C//PVDF-Sn@Zn	2000	700	/	70.3	*Nat. Commun.* **2023**, *14*, 641.[9]
FAG@Zn||MnO_2	2000	2000	100	/	*ACS Nano* **2023**, *17*, 21893.[12]
PI-DT-COF@Zn||MnO_2	2000	1600	89.8	84.1	*Angew. Chem. Int. Ed.* **2024**, e202403918.[14]
PVB@Zn||MnO_2	850	1500	/	86.6	*Adv. Funct. Mater.* **2020**, *30*, 2001263.[20]
NFZP@Zn||V_2O_3	800	500	/	72.8	*Energy Stor. Mater.* **2022**, *47*, 491.[21]
NFSS@Zn||MnO_2	3000	1000	115.7	71.8	*Adv. Sci.* **2023**, *10*, 2303343.[22]
NB@Zn||MnO_2	2000	1000	96.7	/	Nano Lett. **2024**, 24, 9137.[25]
PVA@SR-$ZnMoO_4$ modified Zn||α-MnO_2	1000	1000	141.7	/	*Energ. Environ. Sci.* **2023**, 16, 275.[26]

HI-SEI@Zn||NaV_3O_8-1.5H_2O	500	1100	180	/	*Adv. Funct. Mater.* **2023**, 34, 2308661.[27]
BTO@Zn||MnO_2	2000	300	/	67	*Nano-Micro Lett.* **2021**, 13, 79.[28]
A-Zn/MnO_2	1000	3000	189	93	ACS Energy Lett. **2021**, 6, 3078.[29]

References:

1. Chang X, Zhou X, Ou X *et al.* Ultrahigh nitrogen doping of carbon nanosheets for high capacity and long cycling potassium ion storage. *Adv Energy Mater* 2019; **9**: 1902672.

2. Guo X, Sun H, Li C *et al.* Defect-engineered Mn3O4/CNTs composites enhancing reaction kinetics for zinc-ions storage performance. *J Energy Chem* 2022; **68**: 538-47.

3. Hohenberg P, Kohn W. Density functional theory (DFT). *Phys Rev* 1964; **136**: B864.

4. Kohn W, Sham LJ. Self-consistent equations including exchange and correlation effects. *Phys Rev* 1965; **140**: A1133.

5. Kresse G, Hafner J. Ab initio molecular dynamics for liquid metals. *Phys Rev B* 1993; **47**: 558.

6. J. P. Perdew, K. Burke, M. Ernzerhof. *Phys Rev Lett* 1997; **78**: 1396.

7. Blöchl PE. Projector augmented-wave method. *Phys Rev B* 1994; **50**: 17953.

8. Sun PX, Zheng YQ, Zhang XY *et al.* Spatial Confinement of Sn/TiO2 Nanoparticles in Hollow Mesoporous Carbon Spheres Opal for Stable Zn Metal Anodes. *Adv Energy Mater* 2024; **14**: 2304138.

9. Cao Q, Gao Y, Pu J *et al.* Gradient design of imprinted anode for stable Zn-ion batteries. *Nat Commun* 2023; **14**: 641.

10. Zhou J, Wu F, Mei Y *et al.* Establishing Thermal Infusion Method for Stable Zinc Metal Anodes in Aqueous Zinc-Ion Batteries. *Adv Mater* 2022; **34**: 2200782.

11. Huang Z, Yang S, Zhang Y *et al.* Ultrathin Reed Membranes: Nature's Intimate

Ion-Regulation Skins Safeguarding Zinc Metal Anodes in Aqueous Batteries. *Adv Energy Mater* 2024; **14**: 2400033.

12. Shi Z, Yang M, Ren Y *et al.* Highly Reversible Zn Anodes Achieved by Enhancing Ion-Transport Kinetics and Modulating Zn (002) Deposition. *ACS Nano* 2023; **17**: 21893-904.

13. Peng H, Fang Y, Wang J *et al.* Constructing fast-ion-conductive disordered interphase for high-performance zinc-ion and zinc-iodine batteries. *Matter* 2022; **5**: 4363-78.

14. Guo C, Huang X, Huang J *et al.* Zigzag Hopping Site Embedded Covalent Organic Frameworks Coating for Zn Anode. *Angew Chem Int Ed* 2024: e202403918.

15. Liu H, Xu Z, Cao B *et al.* Marangoni-Driven Self-Assembly MXene As Functional Membrane Enables Dendrite-Free and Flexible Zinc–Iodine Pouch Cells. *Adv Energy Mater* 2024: 2400318.

16. Deng C, Xie X, Han J *et al.* A Sieve-Functional and Uniform-Porous Kaolin Layer toward Stable Zinc Metal Anode. *Adv Funct Mater* 2020; **30**: 2000599.

17. Xin Y, Qi J, Xie H *et al.* 3D Ternary Alloy Artificial Interphase Toward Ultra-Stable and Dendrite-Free Aqueous Zinc Batteries. *Adv Funct Mater* 2024: 2403222.

18. Zhang Q, Luan J, Huang X *et al.* Revealing the role of crystal orientation of protective layers for stable zinc anode. *Nat Commun* 2020; **11**: 3961.

19. Hao J, Li B, Li X *et al.* An in-depth study of Zn metal surface chemistry for advanced aqueous Zn-ion batteries. *Adv Mater* 2020; **32**: 2003021.

20. Hao J, Li X, Zhang S *et al.* Designing Dendrite-Free Zinc Anodes for Advanced

Aqueous Zinc Batteries. *Adv Funct Mater* 2020; **30**: 2001263.

21. Wang S, Yang Z, Chen B *et al.* A highly reversible, dendrite-free zinc metal anodes enabled by a dual-layered interface. *Energy Storage Mater* 2022; **47**: 491-99.

22. Duan J, Dong J, Cao R *et al.* Regulated Zn Plating and Stripping by a Multifunctional Polymer-Alloy Interphase Layer for Stable Zn Metal Anode. *Adv Sci* 2023; **10**: 2303343.

23. Cui Y, Zhao Q, Wu X *et al.* An Interface-Bridged Organic-Inorganic Layer that Suppresses Dendrite Formation and Side Reactions for Ultra-Long-Life Aqueous Zinc Metal Anodes. *Angew Chem Int Ed* 2020; **59**: 16594-601.

24. Zhang R, Feng Y, Ni Y *et al.* Bifunctional Interphase with Target-Distributed Desolvation Sites and Directionally Depositional Ion Flux for Sustainable Zinc Anode. *Angew Chem Int Ed* 2023; **62**: e202304503.

25. Peng Z, Yan H, Zhang Q *et al.* Stabilizing Zinc Anode through Ion Selection Sieving for Aqueous Zn-Ion Batteries. *Nano Lett* 2024; **24**: 9137-46.

26. Chen A, Zhao C, Gao J *et al.* Multifunctional SEI-like structure coating stabilizing Zn anodes at a large current and capacity. *Energ Environ Sci* 2023; **16**: 275-84.

27. Xu X, Li S, Yan H *et al.* A Universal Superhydrophobic-Ionophilic Interfacial Strategy for Cycling Stable Aqueous Zinc Metal Electrodes under Low Current Density. *Adv Funct Mater* 2023; **34**: 2308661.

28. Wu K, Yi J, Liu X *et al.* Regulating Zn Deposition via an Artificial Solid-Electrolyte Interface with Aligned Dipoles for Long Life Zn Anode. *Nano-Micro Lett* 2021; **13**: 79.

29. Park SH, Byeon SY, Park J-H *et al.* Insight into the Critical Role of Surface Hydrophilicity for Dendrite-Free Zinc Metal Anodes. *ACS Energy Lett* 2021; **6**: 3078-85.
