# Supplementary figures and images for: Non-coordinating charge transfer enables ultrafast desolvation of hydrated zinc ions in the outer Helmholtz layer for stable aqueous Zn metal batteries
Source: Natl Sci Rev. 2025 Feb 22;12(4):nwaf070. doi: 10.1093/nsr/nwaf070 (PMC11960093; doi:10.1093/nsr/nwaf070)

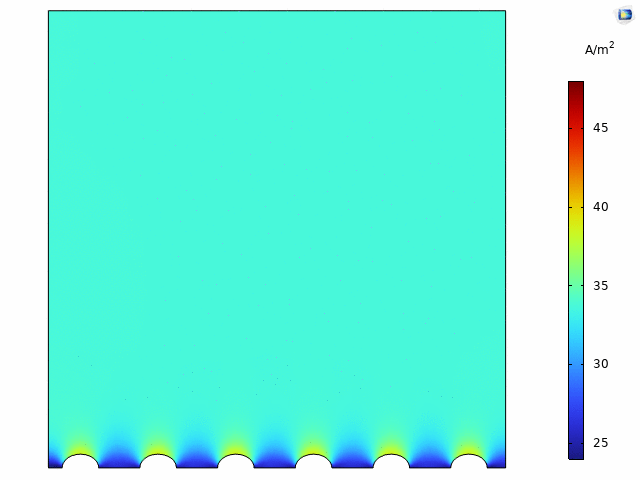

Supplement: nwaf070_Supplemental_Files [file nwaf070_supplemental_files.zip › GIF_1.______Current_density_of_bare_Zn_electrode.gif]

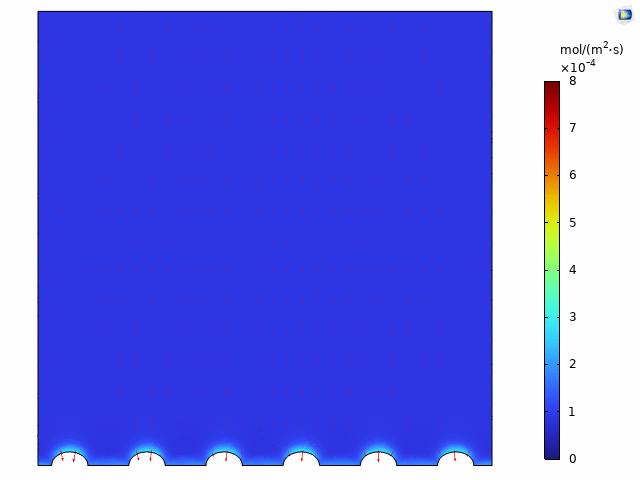

Supplement: nwaf070_Supplemental_Files [file nwaf070_supplemental_files.zip › GIF_2.______Zinc_ions_flux_of_bare_Zn_electrode.gif]

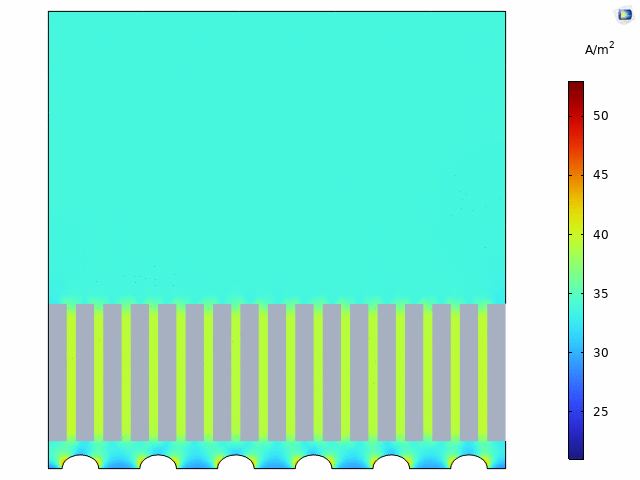

Supplement: nwaf070_Supplemental_Files [file nwaf070_supplemental_files.zip › GIF_3._______Current_density_of_NC-Nafion@Zn_elecetrode.gif]

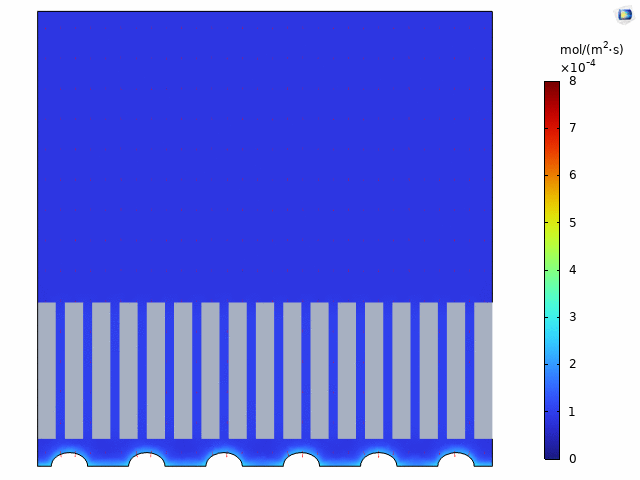

Supplement: nwaf070_Supplemental_Files [file nwaf070_supplemental_files.zip › GIF_4.______Zinc_ions_flux_of_NC-Nafion@Zn_elecetrode.gif]
